# Prevalence of pain 6 months after surgery: a prospective observational study

**DOI:** 10.1186/s12871-016-0261-7

**Published:** 2016-10-10

**Authors:** Rita Laufenberg-Feldmann, Bernd Kappis, Susanne Mauff, Irene Schmidtmann, Marion Ferner

**Affiliations:** 1 Department of Anaesthesiology, University Medical Center of the Johannes Gutenberg University Mainz, Langenbeckstrasse 1, D-55131 Mainz, Germany; 2 Institute for Medical Biostatistics, Epidemiology and Informatics (IMBEI), University Medical Center of the Johannes Gutenberg University Mainz, Langenbeckstrasse 1, D-55131 Mainz, Germany

**Keywords:** Postoperative pain, Analgesics, Chronic pain, Outcome

## Abstract

**Background:**

Pain after surgery is a major issue for patient discomfort and often associated with delayed recovery. The aim of the present study was to evaluate the prevalence of pain and requirement for analgesics up to 6 months after elective surgery, independent if new pain symptoms occurred after surgery or if preoperative pain persisted in the postoperative period.

**Methods:**

A prospective observational single center cohort study was conducted between January 2012 and August 2013. Eligible patients were scheduled to undergo elective surgical interventions including joint (hip, knee arthroplasty), back (nucleotomy, spondylodesis), or urological surgery (cystectomy, prostatectomy, nephrectomy). Pain was assessed on an 11-point numerical rating scale (NRS) before, on postoperative day 2 and 6 months after surgery. Clinical information was collected with structured questionnaires and by telephone interview.

**Results:**

Six hundred and forty-four patients gave informed consent, including 54.4 % men (mean age 62.2, SD 14.3). Higher preoperative pain scores were found in patients undergoing joint (mean 7.6; 95 % confidence interval [CI]: 7.2–8.0) and back surgery (mean 7.1, CI: 6.8–7.5) than in patients prior to urological surgery (mean 2.3; CI: 1.8–2.8). After 6 months, about 50 % of patients after joint or back surgery indicated pain levels ≥3/10, compared to 15.9 % of patients after urological surgery (*p* < .001). 35.3 % of the patients after joint surgery and 41.3 % after back surgery still use pain medication 6 months postoperatively, in contrast to 7.3 % of patients after urological surgery. 13.6 % of patients who underwent back surgery indicated the regular intake of opioids.

**Conclusions:**

Our results reveal that a significant percentage of patients undergoing procedures in joint or back surgery still need pain medication up to 6 months postoperatively due to ongoing pain symptoms. Improved monitoring of pain management is warranted, especially after discharge from hospital, to improve long-term results.

**Trial registration:**

Clinicaltrials.gov (Identifier: NCT01488617); date of registration December 6th 2011.

**Electronic supplementary material:**

The online version of this article (doi:10.1186/s12871-016-0261-7) contains supplementary material, which is available to authorized users.

## Background

Postsurgical pain is a major issue for patient discomfort associated with delayed recovery and prolonged hospital stay [1, 2] and represents a major, largely unrecognized clinical problem [3]. The intensity of acute postoperative pain increases the risk of developing persistent pain [3] and contributes to postoperative morbidity and mortality [4]. Nerve spare surgical techniques are intended to reduce the risk for neuropathic pain which has been identified as an important cause of long-term postsurgical pain, as well as effective early treatment of postoperative pain. In the last two decades guidelines on the management of postoperative pain [5], acute pain services, minimal-invasive surgery and differentiated analgesic therapies have been implemented and evaluated in a national survey in Germany [6]. Multimodal techniques with systemic analgesics should be applied considering the risks and benefits for the individual patient according to Practice Guidelines for Acute Pain Management in the Perioperative Setting [7].

However, the persistence of pain after surgery may lead to functional impairment with consecutive disability and immobility and affect the patients’ quality of life.

Adequate pain management helps to prevent or to diminish postoperative complications, such as respiratory or cardiovascular problems, or postoperative delirium [8–10]. Prevalence and predictors of acute postoperative pain have already been examined [11–16]. There is still a paucity of data to determine the influence of surgical procedure and postoperative pain treatment on long-term pain-related outcome, particularly in patients with preoperative chronic pain.

The aim of this prospective study was to explore the prevalence of pain and requirement for analgesics up to 6 months after elective surgery considering different procedures in joint, back and urological surgery.

## Methods

**Fig. 1:**
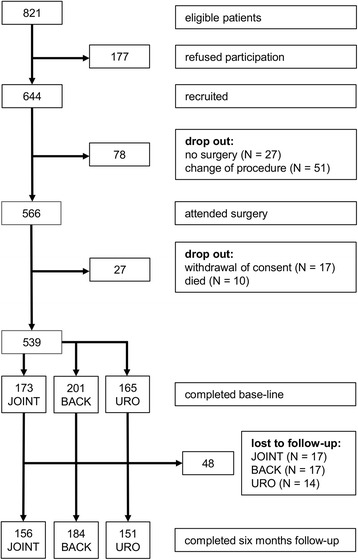
CONSORT-diagram

This single center clinical study was conducted at the Department of Anaesthesiology at the University Medical Center of the Johannes Gutenberg University Mainz, Germany. Data presented here are a subset of a larger data set evaluating outcome variables after major surgery (MOPS-study, Mainz Outcome Predictor Study). Ethical approval for this study [Ethical Committee N° 837.519.11 (8061)] was provided on 3rd January 2012 by the Regional Ethics Committee of Rhineland-Palatinate, Mainz, Germany. The study was registered in clinicaltrials.gov (Identifier: NCT01488617). Written informed consent was obtained from all patients. From January 2012 to August 2013 a total of 821 patients undergoing elective surgery were eligible, from whom 644 (78.4 %) agreed to participate (dropout reasons see Fig. 1, CONSORT-diagram). Surgical interventions included joint surgery (total knee or hip replacement), back surgery (spinal cord decompression with discectomy or hemilaminectomy), and urological surgery (cystectomy, nephrectomy, radical prostatectomy). Urological surgery was predominantly due to cancer.

Eligible patients were aged ≥ 18 years, were able to read, understand German language and were capable to complete questionnaires.

During preoperative consultation prior to surgery, patients completed a set of questionnaires including socio-demographic and pain variables. Pain intensity was assessed on a numerical rating scale (NRS 0–10 with 0 = no pain and 10 = worst possible pain) before surgery, on the second postoperative day, and 6 months after surgery. Pain duration like chronic preoperative pain was defined as pain ≥3/10 NRS for at least 6 months. Pain at 6 months after surgery was defined as at least 3/10 on a NRS, corresponding to moderate pain with a potential impact on physical or emotional functioning [17]. Clinical information was collected from medical records and obtained with structured questionnaires and by telephone interviews in the follow-up period at 6 months after surgery.

Intraoperative analgesia was obtained with a combination of general anaesthesia and if indicated epidural anaesthesia by a continuous epidural infusion of bupivacaine 0.125 % with fentanyl 2 μg/ml and postoperative supplemented with patient controlled epidural analgesia (PCEA).

Standard postoperative pain treatment in the post anaesthesia care unit (PACU) for patients without epidural catheter consisted of patient controlled intravenous analgesia (PCIA) with piritramide (bolus doses of 1.5 mg, lockout time 10 min). In addition, patients with PCIA received non-opioids (e.g. intermittent paracetamol IV every 6 h, metamizole IV 5 g/24 h continuous or NSAIDs). Patients undergoing back surgery received a standard analgesic regimen with non-opioids, supplemented with opioids (15 mg piritramide subcutaneously), if indicated.

On the normal ward, pain treatment is provided and customized twice daily by an acute pain unit, consisting of anaesthesiologists and nursing staff.

### Statistical analysis

As in this study descriptive data, i.e. prevalences, were analyzed, a formal power calculation was not required. Assuming a prevalence of 40 % for pain 6 months after surgery, recruitment of *N* = 145 participants in each surgery group to obtain 95 % confidence intervals of +/− 8 % was required. Descriptive statistics are expressed as absolute and relative frequencies (%) or mean (SD). Differences between groups were examined using parametric Student’s *t*-test or appropriate nonparametric tests (e.g. Kruskal-Wallis Test). Spearman rank correlation (r) was used to examine the association between metric variables. Patients with (NRS ≥3) and without clinically relevant pain at 6 months were compared using cross tabulation and chi-square-tests.

All analyses were performed using IBM SPSS Statistics for Windows, Version 22 (Released 2013. IBM SPSS Statistics for Windows, Armonk, NY: IBM Corp.). *P*-value < .05 was considered statistically significant. No adjustment for multiple testing was performed, therefore only the local significance level α = 0.05 is kept.

## Results

### Patients

From January 2012 to August 2013 644 patients agreed to participate, from which 105 patients had to be withdrawn during the further course of the study for different reasons (Fig. 1, CONSORT diagram). Data of 539 patients were entered in the per-protocol analysis, thereof 173 patients underwent joint surgery, 201 underwent back surgery, and 165 patients underwent urological surgery. Mean follow-up time was 25.8 weeks (SD = 0.8 weeks).

### Demographic and clinical data

Demographic and clinical data are shown in Table 1 for each surgery group.

**Table 1:** Demographic and clinical data

	Joint	Back	Uro	*p*
*N*	173	201	165	
Age (mean, SD)	65.8 (12.9)	59.0 (16.1)	62.4 (12.2)	<.001
Age range	18.0–85.7	18.4–86.9	18.7–82.1	
Female (N, %)	102 (59.0)	103 (51.2)	41 (24.8)	<.001
Without partnership (N, %)	68 (39.3)	57 (28.4)	34 (20.6)	
Educational level				.002
Low (%)	67.1	48.9	47.8	
Medium (%)	22.0	30.0	22.6	
High (%)	11.0	21.1	29.6	
BMI (mean, SD)	30.4 (6.5)	28.0 (4.9)	27.1 (4.6)	<.001
ASA				.001
ASA 1 (%)	1.7	6.5	3.0	
ASA 2 (%)	45.1	50.7	65.2	
ASA 3 (%)	51.4	41.3	30.5	
ASA 4 (%)	1.7	1.5	1.2	
Type of Anaesthesia				<.001
General anaesthesia (%)	48.0	100	38.8	
Regional anaesthesia (%)	39.9	-	-	
Combined general + regional anaesthesia (%)	12.1	-	61.2	
Duration of surgery [Min.] (mean, SD)	123 (53)	152 (85)	273 (145)	<.001
Range	41–438	26–480	59–652	

*ASA* American Society of Anesthesiologists physical status classification system, *BMI* Body mass index

### Surgical interventions

Patients underwent different interventions in orthopedic, neurosurgical and urological surgery. Joint surgery (*N* = 173) included predominantly hip prosthesis (49.7 %), knee prosthesis (48.6 %), shoulder prosthesis (1.7 %). Back surgery (*N* = 201) included discectomy or hemilaminectomy (77.6 %), spondylodesis (20.9 %), and others (1.5 %). Urological surgery comprised (*N* = 165), patients with cystectomy (31.5 %, with continent or incontinent urinary diversion), nephrectomy (27.3 %), radical prostatectomy (38.2 %), and others (3 %).

Urological surgery was mainly due to cancer (86 %), compared to 1.7 % in joint and 2.5 % in back surgery.

### Pain and pain medication

**Fig. 2:**
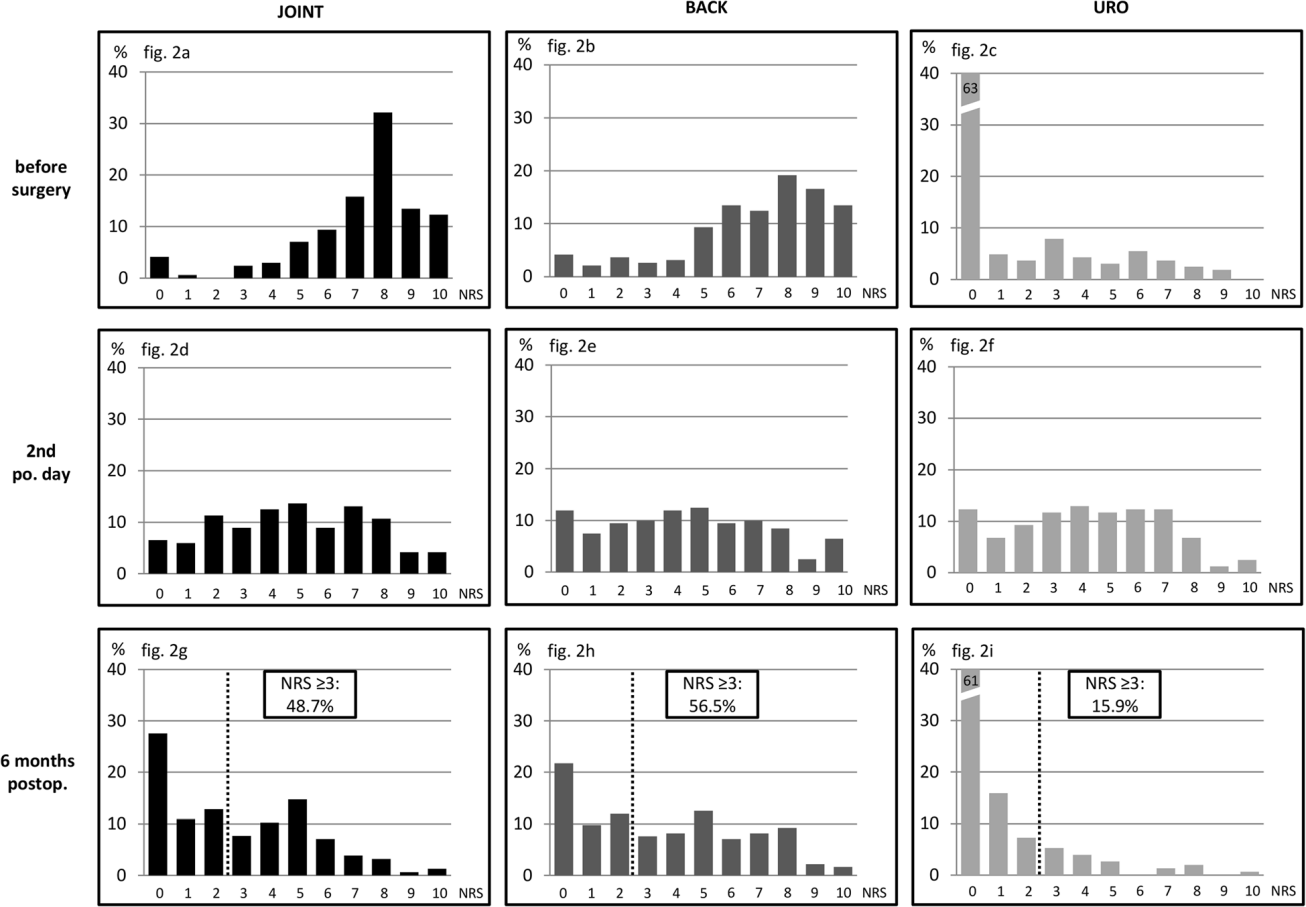
Distribution of pain ratings at three different time points/ surgery groups. The bars show relative frequencies of patients indicating the corresponding ratings on the numerical rating scale (NRS). Patients undergoing joint surgery are presented in *black* (**a**, **d**, and **g**), patients undergoing back surgery in *grey* (**b**, **e**, and **h**), and patients with urological operations in *light grey* (**c**, **f**, and **i**). The *small dotted line* in **g**, **h**, and **i** indicates the proportion of patients quoting pain of “3” and higher

Pain data are presented in Table 2 and in Fig. 2. Patients undergoing joint and back surgery had significantly higher preoperative pain scores than patients prior to urological surgery (Fig. 2a, b, and c). Pain ratings did not differ significantly between the groups in the early postoperative period (Fig. 2d, e, and f). After 6 months significant differences between the three surgery groups became apparent (Fig. 2g, h, and i). Patients after back surgery indicated the highest pain scores, followed by joint surgery. After urological surgery patients reported the lowest pain levels at that time.

**Table 2:** Pain ratings and use of analgesics

	Joint	Back	Uro	p*
Chronic preoperative pain^a^ [%]	79.2	43.8	18.8	<.001
Preoperative (mean, SD)^b^	7.2 (2.3)	6.8 (2.6)	1.6 (2.6)	<.001
2 days postoperative (mean, SD)^b^	4.8 (2.7)	4.5 (2.9)	4.2 (2.7)	.136
6 months postoperative (mean, SD)^b^	2.9 (2.6)	3.6 (2.9)	1.1 (1.9)	<.001
Analgesics at endpoint (%)^c^	35.3	41.3	7.3	<.001
Non-Opioids at endpoint (%)^c^	31.3	34.4	6.0	<.001
Opioids at endpoint (%)^c^	8.7	13.6	2.0	.001

*Kruskal-Wallis Test, Chi-Square Test, resp

^a^NRS ≥ 3 and duration at least six months prior to surgery

^b^Pain ratings during movement on a Numeric Rating Scale (NRS) 0–10

^c^Regular intake of analgesics 6 months after surgery

### Sex differences

Considering the entire sample, women indicated higher mean pain scores than men both preoperatively (6.5 vs. 4.3, *p* < .*001*) and 6 months after surgery (3.0 vs. 2.3, *p* = .*005*), while there were no differences on the second postoperative day. Subdivided into surgery groups, the preoperative gender-specific differences were not confirmed in the joint surgery group (*p* = .*345*). Also in the catamnesis, no gender-specific differences were observed in patients undergoing joint (*p* = .*987*) and back surgery (*p* = .*629*) indicating similar pain levels in men and women in these subsamples, respectively.

With regard to preoperative pain status, there were differences between all groups. Almost 80 % of the patients undergoing joint and 44 % of the patients undergoing back surgery suffered from chronic preoperative pain, while this applied only to a quarter of the patients prior to urological surgery.

Six months after surgery, 35.3 % of the patients after joint surgery and 41.3 % after back surgery still used analgesics (Table 2), in contrast to 7.3 % of patients after urological surgery. 13.6 % of patients who underwent back surgery indicated the regular intake of opioids.

41.5 % of the patients in our sample experienced clinically relevant pain 6 months after surgery, with different rates in each surgery group (Fig. 2g, h, and i). After joint and back surgery about every second patient indicated pain levels ≥3/10 at that time. However, only 15.9 % of patients after urological surgery reported similar pain levels *(p* < .*001)*.

Table 3 shows comparisons of different clinical variables between patients with and without clinically relevant pain in each surgery group 6 months postoperatively.

**Table 3:** Comparison of clinical variables between patients with and without pain 6 months after surgery

	Joint pain	Joint no pain	p*	Back pain	Back no pain	p*	Uro pain	Uro no pain	p*
N (%)	76 (48.7 %)	80 (51.3 %)		104 (56.5 %)	80 (43.5 %)		24 (15.9 %)	127 (84.1 %)	
Female (%)	56.6	60.0	.39	51.9	51.3	.52	50	22	.006
Age (mean, SD)	65.3 (12.4)	66.6 (13.3)	.40	61.8 (14.9)	60.0 (16.6)	.062	60.5 (11.5)	62.8 (11.7)	.30
BMI (mean, SD)	30.9 (5.5)	30.0 (7.3)	.053	28.9 (4.7)	27.1 (5.1)	.004	26.5 (4.6)	27.4 (4.5)	.34
Preoperative Pain Intensity NRS ≥3 (%)	94.7	92.5	.41	92.3	73.8	.001	54.2	22.8	.003
Chronic Preoperative Pain (%)^a^	85.5	75.0	.074	54.8	30.0	.001	33.3	15.7	.046
Duration of Surgery [minutes] (mean, SD)	124 (56)	123 (53)	.74	154 (86)	147 (79)	.64	275 (136)	268 (147)	.62

*NRS* Numeric Rating Scale

*Kruskal-Wallis Test, Chi-Square Test, resp

^a^NRS ≥ 3 and duration at least six months prior to surgery

Significant differences between men and women were observed in patients undergoing urological surgery. First, there were only 40 women (26.5 %) in this group of 151 patients. Furthermore, 63 male patients underwent radical prostatectomy (RPX), being associated with low postoperative pain scores (*N* = 63, mean = 0.6, SD = 0.2). Considering surgical interventions that were performed in both men and women (nephrectomies and cystectomies), the comparison of pain scores at 6 months revealed no significant differences (men: *N* = 52, mean = 1.1, SD = 2.0; women: *N* = 40, mean = 1.7, SD = 2.4, *p* = .20). This also applies for the percentage of clinically relevant pain 6 months after surgery (17.3 % in men vs. 30.0 % in women, *p* = .21).

BMI levels were slightly higher in patients who indicated pain compared to patients without pain, however, differences in BMI levels did not reach statistical significance (joint surgery: mean 30.9 vs 30.0, *p* = *0.053*; back surgery: mean 28.9 vs 27.1, *p* = *0.004*).

Preoperative pain variables (i.e. pain intensity and duration) differed significantly between patients 6 month after surgery. Patients who suffered from pain at 6 months were more likely to have reported preoperative pain intensity of ≥ 3/10 NRS or to suffer from preoperative pain for more than 6 months prior to surgery.

Considering the duration of surgery, no significant differences between patients with or without pain were noticed between any of the surgery groups (Table 3).

## Discussion

The results of the present study demonstrate that a significant number of patients report persistent pain and intake of analgesic medication, even opioids, up to 6 months after surgery.

Considering the different surgery groups, more than 50 % of patients after back and joint surgery indicated clinically relevant pain 6 months after surgery. Patients undergoing joint or lumbar spine surgery for degenerative conditions often have poor outcomes following the surgical intervention, with up to 40 % reporting residual chronic pain [1, 18]. Many studies on treatment outcome focus on pain ratings in the acute postoperative phase after different surgery [12, 19, 20], or have investigated prevalence and etiology of persistent postsurgical pain (PPSP) [3, 21–23]. According to the definition by Macrae [24] for persistent postsurgical pain the following criteria should be fulfilled: pain after surgical procedure, pain duration from at least 2 months, excluding other conditions and exclusion from a preexisting problem. Particularly conditions in which surgery escalates pre-existing pain symptoms have to be explored carefully.

Especially in patients with degenerative diseases of joint or vertebral column, the patients’ burden of pain often triggers the indication for surgery. Therefore, these patients do not meet the above-mentioned criteria for persistent post-surgical pain. In contrast, in patients undergoing major surgery due to urological tumors, preoperative pain is not a leading symptom.

We decided to follow-up patients up to 6 months after surgery assuming that postoperative rehabilitation programs were completed at that time and prolonged inflammatory processes would have been subsided [24]. Patients in our study who underwent back (or joint) surgery, experienced higher preoperative and postoperative pain levels compared to patients in other studies undergoing surgery of the vertebral column or knee replacement surgery [18, 25].

With regard to postoperative pain, patients with renal cancer [21] or with prostate cancer [26–28] indicate low postoperative pain in the catamnesis, which is in agreement with our data. However, self-reported pain levels after urological surgery were lower than in a study evaluating pain in patients after radical prostatectomy, with a significant proportion of patients complaining about pain 3 and 6 months after surgery [26].

In contrast to other studies, we evaluated prevalence and intensity of pain 6 months after surgery in combination with the need for pain medication. We assumed that after joint and back surgery, pain symptoms would improve continuously so that a regular intake of analgesics could be avoided. However pain intensity and pain duration differed between surgical procedures in our study. Especially patients undergoing back or joint surgery appeared to be impaired due to pain up to 6 months postoperatively. Consequently, in our sample, about 40 % of the patients after back surgery reported the intake of analgesics 6 months post-surgery, and 13.6 % of these patients even indicated the intake of opioids on a regular basis.

These results are unexpected and show an unsatisfying result of the surgical intervention in terms of pain reduction. Moreover, the long-term intake of opioids in non-cancer pain is not supported by recent clinical practice guidelines [29], since opioids have significant side effects and their analgesic effect is not superior to non-opioids.

Possible explanations for the prolonged intake of analgesics after surgery may be an uncritical prescription of opioids or uncontrolled self-medication with analgesics. In patients undergoing spine surgery, psychiatric co-morbidity may be a risk factor for continued postoperative opioid intake, especially if opioids were already used prior to surgery [30]. Also in patients with total knee replacement the preoperative use of opioids may increase the risk for post-operative pain during the first post-operative week with consecutive higher opioid consumption [31].

More effective pain management strategies already before and after surgery may provide improved surgical outcomes. This may contribute to a better quality of life [32] and may help to prevent persistent pain, whereas insufficient pain management is associated with poorer recovery, and worse quality of life, also on long-term follow-up [16].

Cognitive and behavioral strategies in combination with individualized postoperative rehabilitation programs may also contribute to reduce the patients’ burden of pain and provide better surgical long-term results. Avoiding fear of movement and decreased physical function has already shown to be an important aspect e.g. in patients following lumbar spine surgery for degenerative conditions [18].

Some limitations of the study should be considered. For the follow-up period, a longer observational time (e.g. up to 1 year) may have been reasonable in order to evaluate patients with a late improvement of pain symptoms. In addition, during the follow-up period no physical examination was performed in order to evaluate the functional improvement after surgery. Therefore, we could not state on functional success of surgery after physical therapy and rehabilitation programs. Furthermore, we did not discriminate whether the patients with urological surgery received a curative or a palliative intervention. However, since these patients reported the lowest pain levels in comparison to the other groups, before and after surgery, this aspect was not further investigated.

## Conclusions

In conclusion, these data reveal that a significant number of patients suffers from pain and need analgesic medication, even opioids, up to 6 months after surgery. The treatment of ongoing pain after surgery is still an important challenge. The need of preventive therapies should be considered for example in patients with neuropathic pain or patients with chronic opioid use. Besides the evaluation of predictive factors for ongoing postoperative pain and the identification of patients at risk, the improvement of postoperative pain management strategies after discharge should be subject of further research.

## Additional file

Additional file 1: Description of variables. Data set includes socio-demographic pain related, anesthesia-related data as well as data on pain medication. (XLSX 85 kb)

## Abbreviations

BMI: Body mass index
IV.: Intravenous
NRS: Numerical rating scale
NSAID: Nonsteroidal anti-inflammatory drugs
PACU: Postanaesthesia care unit
PCEA: Patient controlled epidural analgesia
PCIA: Patient controlled intravenous analgesia
PPSP: Persistent postsurgical pain
RPX: Radical prostatectomy
SD: Standard deviation
